# Implications of an Absolute Simultaneity Theory for Cosmology and Universe Acceleration

**DOI:** 10.1371/journal.pone.0115550

**Published:** 2014-12-23

**Authors:** Edward T. Kipreos

**Affiliations:** University of Georgia, Athens, GA 30602, United States of America; University of Pécs Medical School, Hungary

## Abstract

An alternate Lorentz transformation, Absolute Lorentz Transformation (ALT), has similar kinematics to special relativity yet maintains absolute simultaneity in the context of a preferred reference frame. In this study, it is shown that ALT is compatible with current experiments to test Lorentz invariance only if the proposed preferred reference frame is locally equivalent to the Earth-centered non-rotating inertial reference frame, with the inference that in an ALT framework, preferred reference frames are associated with centers of gravitational mass. Applying this theoretical framework to cosmological data produces a scenario of universal time contraction in the past. In this scenario, past time contraction would be associated with increased levels of blueshifted light emissions from cosmological objects when viewed from our current perspective. The observation that distant Type Ia supernovae are dimmer than predicted by linear Hubble expansion currently provides the most direct evidence for an accelerating universe. Adjusting for the effects of time contraction on a redshift–distance modulus diagram produces a linear distribution of supernovae over the full redshift spectrum that is consistent with a non-accelerating universe.

## Introduction

The Absolute Lorentz Transformation (ALT) is an alternate Lorentz transformation that has similar kinematics to special relativity (SR), but is distinct in describing absolute simultaneity and invoking a preferred reference frame (PRF) relative to which time dilation and length contraction occur in a directional manner [1]–[3]. The key insights in this study are the following. ALT is compatible with current experimental data if it is embedded in the theoretical framework that PRFs are locally associated with centers of gravitational mass. Experimental strategies that focus on light speed anisotropies and time dilation in relation to local centers of gravitational mass can distinguish between the ALT framework and SR. The ALT framework is more compatible with the interpretation of cosmological redshift as kinematic Doppler shift than with the conventional interpretation of photons being modified directly by the expansion of space. Combining the ALT framework with the kinematic interpretation of cosmological redshift creates a scenario in which Hubble expansion is linked to time dilation on a universal scale. Analysis of Type Ia supernovae in the context of this scenario provides an alternate explanation for the reduced luminosity of high redshift Type Ia supernovae that does not invoke an acceleration in the rate of universe expansion.

### The Absolute Lorentz Transformation

The Lorentz transformation equations were first described by J. Larmor [4], H.A. Lorentz [5], and J.H. Poincaré [6] as directional transformations for objects in motion relative to the ether as a PRF. Einstein's 1905 paper describing SR independently derived the Lorentz transformation with the stipulation that all inertial reference frames are equivalent [7]. In SR, Lorentz transformations are reciprocal, and occur in the context of differential simultaneity.

R. Mansouri & R.U. Sexl created a widely-used test theory for SR [3]. The Mansouri & Sexl (MS) test theory describes transformations between an “ether frame” Σ (with space-time coordinates *X, T*) and an inertial reference frame S (with space-time coordinates *x, t*). The transformation equations include arbitrary functions of velocity: 1/*a*(*v*) is the time dilation factor; *b*(*v*) is the length contraction factor; and 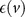 is determined by the convention of clock synchronization. The MS test theory is described in an unconventional format in which *t* is calculated relative to *T* and *x* (rather than *T* and *X*). 
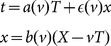
(1)


In the MS test theory convention, the Lorentz transformation has: 1/*a*(*v*)  =  *b*(*v*)  = 1/(1 - *v* ^2^/*c* ^2^)^1/2^; and 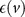  =  -*v*/*c* ^2^ [3]. 
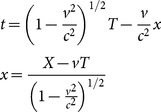
(2)


A. Eagle, F.R. Tangherlini, and Mansouri & Sexl described a modified Lorentz transformation that maintains absolute simultaneity [1]–[3], [8]. Tangherlini referred to the transformation as ALT [2]. ALT is represented in the MS test theory convention as: 1/*a*(*v*)  =  *b*(*v*)  = 1/(1 - *v* ^2^/*c* ^2^)^1/2^; and 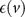  = 0. 
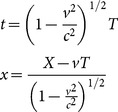
(3)


SR and ALT have similar kinematics. The form of the Lorentz transformation equation that is generally used in experimental settings to calculate time dilation is identical to the ALT time dilation equation. As described in Einstein's 1905 paper [7], the Lorentz time dilation equation *t′*  =  (*t* - *vx/c* ^2^)/(1 - *v* ^2^/*c* ^2^)^1/2^ with the value *x* =  *vt* produces *t′*  =  *t*(1 - *v* ^2^/*c* ^2^)^1/2^, which is the ALT equation (3). Mansouri & Sexl noted that ALT is the very relation one would write down if one has to formulate a theory in which rods shrink by a factor (1 - *v* ^2^/*c* ^2^ *)* ^1/2^ and clocks are slow by a factor (1 - *v* ^2^/*c* ^2^ *)* ^1/2^ when moving with respect to a PRF [3].

### Differences between ALT and SR

ALT differs from SR in several respects. ALT maintains absolute simultaneity for all observers, while SR implies local differential simultaneity [2], [3]. The corollary to this is that SR maintains light speed isotropy between inertial reference frames, while ALT implies anisotropies in the one-way speed of light, although the two-way speed of light for ALT is *c* [3], [9]. The two theories also differ in that time dilation between inertial reference frames is reciprocal for SR and directional for ALT [2], [3]. With directional time dilation, observers in a PRF will observe that clocks moving relative to the PRF run slower, while observers in non-PRF reference frames will observe that clocks in the PRF run faster (i.e., exhibit time contraction) [2], [3]. The directional time dilation specified by ALT is absolute, and clocks can be compared directly for time differences that reflect the extent of time dilation. Further, time dilation in the two theories is calculated relative to different reference frames [2], [3]. In SR, Lorentz transformations are calculated reciprocally using the relative velocity between inertial reference frames. In contrast, ALT is calculated relative to the PRF for each observer.

SR does not preclude an absolute reference frame. Lorentz and Poincaré believed in the existence of an absolute reference frame in the context of the Lorentz transformation [6], [10]. However, unlike ALT, SR cannot distinguish between an absolute reference frame and other inertial reference frames. This is because SR predicts equivalent, reciprocal time dilation and length contraction between any two inertial reference frames, including a potential absolute reference frame.

Throughout the remainder of this study, ‘PRF’ will not be used in the sense of an absolute reference frame, but rather in the broader sense to refer to any reference frame relative to which Lorentz/ALT transformations occur in a directional manner.

### Evidence supporting directional time dilation relative to the ECI

Experimental evidence from Hafele & Keating indicates that the Earth-centered non-rotating inertial reference frame (ECI) can act as a local reference frame to direct time dilation (i.e., a PRF in the broader sense). In their experiment, atomic clocks were flown in airplanes eastward and westward around the Earth, and time dilation was calculated relative to the ECI [11], [12]. Flying eastward, in the direction of the Earth's rotation, increased the speed of the airplane relative to the non-rotating ECI; while flying westward, in the direction opposite of the Earth's rotation, produced a slower speed relative to the ECI. The Lorentz/ALT time dilation formula was applied to the velocity of the ground-based clocks relative to the ECI and to velocities of the flying clocks relative to the ECI in order to calculate the extent of time dilation [11]. The flying clocks recorded the expected loss of time on the eastward flight, and the expected gain of time on the westward flight when compared to the ground-based clocks. More accurate repetitions of the Hafele & Keating experiment have similarly obtained the expected time dilations for movements relative to the ECI [13]–[15].

In the Hafele & Keating experiment, the time dilation was absolute and directional, as the flying and ground-based clocks showed different elapsed times when brought together for side-by-side comparisons. Hafele & Keating suggested that the directional time dilation arose within the context of SR because objects in non-inertial reference frames experience directional time dilation relative to inertial reference frames [11]. However, the section below will show that absolute directional time dilation is also observed between inertial reference frames.

Satellites of the global positioning system (GPS) are in inertial reference frames because they are in free-fall orbits around the Earth, similar to the inertial reference frame of the ECI that arises from its free-fall orbit around the Sun. It is well established that the ECI functions as a PRF for GPS satellites, with the satellites experiencing directional time dilation based on their velocity relative to the ECI [16]. Clocks on GPS satellites undergo time dilation of ∼7 µs per day relative to the Earth's surface, which is calculated by applying the Lorentz/ALT time dilation formula independently to the speed of the satellite relative to the ECI and to the speed of the Earth's surface relative to the ECI [17]. Correcting for the Lorentz/ALT time dilation is essential for proper positioning in the GPS system, as the 7 µs/day difference translates to a localization error of 2.1 km per day [17]. The Sagnac effect, which is important for the communication of GPS satellites with rotating ground-based receivers, is irrelevant to the time dilation experienced by the satellites as they move relative to the non-rotating ECI [16]. The communication between GPS satellites and ground-based clocks continuously reveals the absolute and directional nature of the time dilation.

### The interpretation of cosmological redshift as kinematic movement

In 1929, Edwin Hubble provided evidence that the recession velocities of galaxies increase linearly with distance, thereby inferring that the Universe is expanding [18]. The Hubble constant, recently estimated to be 73±2 km/s/Mpc [19], defines the rate at which objects separate from each other with increasing cosmological distance.

Cosmological redshift (*z*) can be correlated with the change in universe scale during expansion [20], [21]. The lengthening of the wavelength of the cosmic microwave background (CMB) (and its consequent cooling) correlates with the cosmic scale factor *a(t)*, 1/(1+*z*) [22]. The conventional interpretation of cosmological redshift is that it arises as the wavelength of photons are lengthened as they traverse through expanding space [23].

Cosmological redshift can be interpreted as kinematic relativistic Doppler shift by a mathematical treatment of transporting the velocity four-vector from the source to the observer [24], and through analyses of Friedman–Lemaître–Robertson–Walker (FLRW) models [25], [26]. While the kinematic interpretation of cosmological redshift is unconventional, it incorporates a well-characterized mechanism, relativistic Doppler shift, and can also explain the lengthening of light wavelengths with universe expansion. Application of the relativistic Doppler shift equation and the relativistic law of addition of velocities to the kinematic motion of cosmological objects produces the same linkage between the cosmic scale factor and changes in wavelength [27]. The kinematic interpretation of redshift therefore provides an alternate explanation for the observed lengthening of wavelength and cooling of the CMB radiation.

## Theoretical Considerations

### Conditions under which ALT is compatible with experimental evidence

There is a large body of published data that shows no violations of Lorentz invariance for experiments carried out on the Earth or in the local Earth environment [28]. These experiments observed the predicted Lorentz time dilations regardless of the Earth's movement, which would be expected to alter the speed of the experimental instrument relative to an external PRF. With ALT, time dilation is calculated using the velocity of the reference frame relative to the PRF, so in a valid ALT scenario, an external PRF would affect time dilation on the Earth as the Earth moved relative to the PRF. Tests of Lorentz invariance often use the MS test theory to provide a lower limit on the confidence of Lorentz invariance [29]. These lower confidence limits are equivalent to increasingly restricting the movement (drift) of a potential PRF relative to the experimental apparatus [30], [31].

Mansouri & Sexl suggested that the CMB frame is the obvious candidate for a possible “ether frame” [3]. However, the CMB cannot be the PRF for a viable ALT, as the movement of the Solar System relative to the CMB (∼368 km/s, [22]) greatly exceeds the allowable PRF drift that is calculated using the MS test theory [30], [31]. Based on the extensive tests of Lorentz invariance that have been carried out on or near the Earth [28], [29], the only viable scenario for ALT is that a PRF must be locally associated with the Earth, in particular, with the ECI.

The requirement for the PRF to be locally centered on the ECI has implications for the concept of the ether. The ether is defined as the medium for the propagation of electromagnetic radiation [32]. The concept of the ether has been considered for more than 100 years, yet during this period, no compelling experimental evidence has supported the existence of a specific medium for the propagation of light. Therefore, the viability of the ether concept is tenuous. The observation of stellar aberration indicates that starlight does not move in the same reference frame as the Earth, and this implies that the ether cannot be dragged/entrained by the Earth [32]. Both ALT and SR have the same formula for the angle of stellar aberration [33]. Therefore, in the scenario of a valid ALT, the ether cannot be equivalent to the PRF because ALT is only compatible with a PRF that is locally centered on the ECI, and yet the ether, if it exists, cannot be locally centered on the ECI.

The observation of directional time dilation relative to the ECI indicates that the ECI functions locally as a PRF (broadly defined). Both the ECI and GPS satellites are in “free fall” inertial reference frames, and yet GPS satellites experience directional time dilation relative to the ECI. This indicates that directional time dilation is not limited to the interaction of non-inertial and inertial reference frames but is also observed between inertial reference frames. It therefore raises the issue of why the ECI functions as a PRF. The force of gravity connects the ECI and the objects that experience directional time dilation as a result of motion relative to the ECI. A plausible hypothesis is that the ECI functions as a PRF because it is the local center of mass with the dominant gravitational field in its local environment. The combination of ALT and PRFs linked to local centers of gravitational mass will be referred to as absolute simultaneity theory (AST).

### Experimental approaches to distinguish SR and AST

Mansouri & Sexl state that there is the impossibility of an ‘experimentum crucis’ that can distinguish between SR and ALT because both have similar kinematics [30]. There are, however, two differences between SR and ALT (in the context of AST) that can be distinguished experimentally.

The first experimentally distinguishable difference between the two theories is that ALT allows anisotropies in the one-way speed of light, while light speed is isotropic with SR [3], [9]. However, the designs of experiments to analyze one-way light speeds have been incapable of detecting the light speed anisotropies predicted by the AST framework. With the exception of a space flight experiment that could not distinguish between potential anisotropies in the speed of light and gravitational effects [34], [35], all of the modern experiments to detect the one-way speed of light have relied on changes in the Earth's movement to alter the speed of the test equipment relative to a potential external PRF [36]–[44]. The null results of these experiments are compatible with the ECI as the PRF, as the movement of the Earth would not alter the location of the test equipment relative to the ECI.

Experimental approaches using one-way light paths have demonstrated that light speeds are anisotropic when measured from the rotating Earth surface; these approaches include the Michelson-Gale experiment [45], [46] as well as other experiments that reveal the Sagnac effect relative to the ECI, including GPS satellite communications [16], [47]. The Sagnac effect is consistent with AST because light is predicted to propagate isotropically only in PRFs, but not in reference frames moving relative to a PRF, such as the rotation of the Earth's surface relative to the non-rotating ECI [3]. The Sagnac effect does not conflict with SR because rotational movements are considered to be exempt from the relativity principle [48]. Therefore, current experiments to analyze the speed of light do not distinguish between the two theories.

It is possible to design experiments that would be capable of detecting light speed anisotropies predicted by AST in the context of a proposed gravitational mass-based PRF moving relative to an inertial reference frame. Consider two observers at rest in the heliocentric reference frame who are separated from each other parallel to and near Earth's orbit. When the Earth is next to the observers, they send light signals between themselves so that the light signals move in the direction of the Earth's orbital motion or opposite to the Earth's motion. Viewed from the ECI perspective, the observers are in an inertial reference frame moving past the ECI, and one observer appears to move toward the light signal sent in the direction of Earth's orbital motion, while the other observer moves away from the light signal sent in the other direction. This situation can be considered analogous to the AST perspective on the Sagnac effect, where observers on the rotating Earth move toward or away from light beams that propagate isotropically in the ECI. Just as observers on the Earth surface or in orbit around the Earth calculate light speed anisotropies when sending light signals among themselves [16], [45]–[47], in an AST framework, the heliocentric observers would similarly experience light speed anisotropies: light sent in the direction of the Earth's motion would appear faster than *c*, and light sent in the direction opposite of the Earth's motion would appear slower than *c*. The same experiment conducted when the Earth was distant from the two heliocentric observers (so that their main gravitational influence becomes the Sun, with which they are at rest) would predict isotropic light speeds within the AST framework. In contrast, SR predicts isotropic light speeds in all situations.

The second experimentally distinguishable difference between the two theories is that AST predicts directional time dilation for inertial reference frames moving relative to a proposed PRF [2], [3], while SR predicts reciprocal time dilation.

Experiments that utilize atomic clocks traveling in inertial reference frames near a proposed gravitational mass-based PRF can be used to probe for differences in time dilation. For example, clocks could be sent past the Earth in the direction of and opposite to the Earth's orbital motion in linear inertial paths. For each clock, the time dilation due to gravitational effects would be calculated and subtracted from the total observed time dilation to determine the time dilation due to motion. This can be accomplished because time dilation due to gravity (calculated using general relativity, GR) and motion (calculated using the Lorentz transformation/ALT) are, in practice, independent and additive [11], [17]. In the proposed experiment, AST predicts that the clock traveling in the direction of PRF motion would experience less time dilation than the clock moving in the direction opposite of PRF motion. This is because the former clock would have a lower velocity relative to the PRF, and the latter clock would have a higher velocity. In contrast, SR does not predict directional time dilation between objects moving in inertial reference frames, and there is no theoretical basis within SR for assigning a different velocity based on the motion of a nearby gravitational mass. The clocks can be considered to be traveling in inertial reference frames because their constant-speed linear trajectories would only be affected to a limited extent by free fall in Earth's gravity, which would also be inertial.

### The application of ALT to cosmological data

Historically, SR has not been used extensively in general relativistic cosmology (GRC). This can be attributed in part to the historical view that Minkowski spacetime applies only in situations devoid of mass and energy [49], and the designation of SR as a limiting case of GR that is only valid in small, local settings [50]. These historical considerations would not apply to ALT, which is not encompassed by Minkowski spacetime or current GRC theories.

The Lorentz transformation/ALT time dilation equation functions robustly in conditions that have classically not been associated with Minkowski spacetime. The Lorentz transformation/ALT equation can accurately calculate the time dilation of objects traveling in non-inertial frames [12]. It can also accurately predict the time dilation of muons traveling in a circular cyclotron using only the speed of the muons as input; and this motion is, by definition, accelerated motion [51]. Further, the Lorentz transformation/ALT equation accurately predicts the time dilation of subatomic particles traveling through Earth's atmosphere [52], which is neither empty nor flat, with densities of matter and curvature of space that are significantly higher than that found in intergalactic space. This wide applicability is consistent with ALT for which there is no theoretical basis to limit its application to inertial reference frames.

### AST implies universal time dilation

The convention in cosmology is to use a comoving universe coordinate system that expands in sync with the Hubble expansion [49]. However, AST implies that PRFs are linked to centers of gravitational mass, which implies that an AST coordinate system would be non-comoving. In a non-comoving coordinate system, the interpretation of cosmological redshift as kinematic relativistic Doppler shift can be applied to objects separating due to Hubble expansion. In this context, higher redshifts linked to Hubble expansion signify increased velocities of separation between observers (at the time the light is received) and cosmological objects (at the time the light was emitted in the past). Thus objects in the present Universe can be interpreted to have increased kinematic velocities relative to objects in the past. The application of ALT to recession velocities would imply that objects in the present Universe experience time dilation relative to objects in the past. Conversely, when viewed from the present, objects in the past would have experienced time contraction.

Time contraction would have effects on both redshift and luminosity. From the vantage point of our present time scale, photons emitted in the past under time-contracted conditions would have been emitted at a faster rate, with blueshifted wavelengths (as the frequency of the emitted light was increased relative to our time scale).

## Results

### Universal time dilation implies a non-accelerating universe

Type Ia supernovae (SNe Ia) function as standard candles, and the analysis of their redshift and luminosity has provided unique insights into universe evolution [20]. The effect of time contraction (TC) on the placement of SNe Ia in a Hubble-type diagram will be analyzed using data from the Supernova Cosmology Project (SCP) Union 2.1 compilation [53], [54].

The relativistic Doppler shift formula is used to calculate the effective recession velocity (*v_er_*) of SNe Ia based on their observed redshifts.



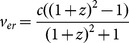
(4)Based on their apparent magnitudes, SNe Ia at high redshift are separating with velocities greater than *c*, as expected for an expansion rate based on the Hubble constant [55]. While the relativistic Doppler shift equation (4) will not produce velocities greater than *c*, it can be used as a conduit between the redshift and time dilation formulas; i.e., it is the effective velocity embedded in the redshift value for time dilation calculations.

The ALT time dilation formula (3) is used to calculate the time-contraction ratio (*TC*), which represents the ratio of the number of time intervals for an object emitting light in the past (Δ*t_e_*) relative to the number of time intervals for an observer in the present (Δ*t_o_*).



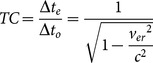
(5)
*TC* increases above 1 as *v_er_* increases, reflecting that at high *v_er_* values, more than one unit of time occurred in the past for every present-day time unit (e.g., for a *v_er_* of 0.6 *c*, 1.25 s elapsed in the past for every 1 s in the present).

**Figure 1:**
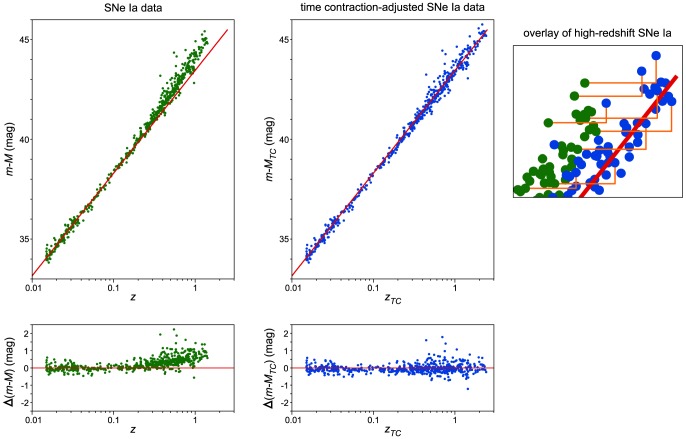
Top: Diagrams of SNe plotted for *z* and *m-M* (green, left) and *z_TC_* and *m-M_TC_* for which redshifts and distance moduli have been adjusted to compensate for increased blueshifted emissions under TC conditions (blue, center). The straight line in each is a linear regression derived using SNe Ia with *z*<0.14. Bottom: Residuals in distance moduli relative to the linear regression line derived using SNe Ia with *z*<0.14. An overlay of high-redshift SNe Ia at increased magnification is shown on the right. Orange lines mark the shift between positions for selected SNe Ia.

Substituting the definition of *v_er_* from equation (4) into equation (5) produces the formula for *TC* in terms of *z*.



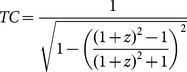
(6)Time contraction on the scale of the Universe is linked to Hubble expansion. A direct link between time contraction and universe expansion can be illustrated by expressing the equation for time contraction (6) in terms of the scale factor *a*(*t*).



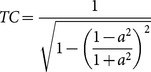
(7)While not widely considered, the normal interpretation of Hubble diagrams has the embedded inference that the positions of SNe Ia reflect their distance and luminosity based on the SNe Ia emitting light at their normal rate (e.g., the redshift value denotes the change in redshift from the observed redshift to the normal emission redshift). The effects of time contraction alter the proper placement of SNe Ia on a plot of redshift and distance modulus. Under a time-contraction scenario, the wavelengths of SNe Ia at higher redshifts were blueshifted at the time of emission. Therefore, the light from these SNe Ia underwent a larger change in wavelength (from blueshift to redshift) than is reported in the Hubble diagram. The total change in *z* value from the time-contracted, blueshifted emission to the observed redshift is given by *z_TC_*, which will be derived below. It is known that:
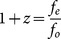
(8)where *f_e_* is the inferred frequency of light emitted and *f_o_* is the frequency of light observed. Rearranging equation (8) gives:



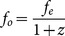
(9)The effect of time contraction increases *f_e_* in equation (8) by the time contraction ratio (*TC*) to give:



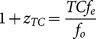
(10)Substituting the value of *f_o_* from equation (9) into equation (10), and simplifying, gives:



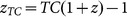
(11)To reflect the larger change in redshift from emission to detection, SNe Ia are shifted to the higher *z_TC_* redshift position (rightward) on the diagram (Fig. 1).

Under time-contraction conditions, the rates of photon emissions for SNe Ia in the past were increased when viewed from our current, time-dilated perspective. To compensate for the increased emission rates, SNe Ia are shifted to higher distance modulus values (upward) on the diagram to reflect the lower level of luminosity that would have occurred if the SNe Ia were emitting at the current (non-time contracted) rate (Fig. 1). This adjustment is necessary because the use of SNe Ia as standard candles inherently requires that all SNe Ia have the same emission rate. The formula for apparent magnitude (*m*) is:
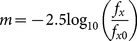
(12)where *f_x_*/*f_x0_* is the observed flux. Multiplying the observed flux by 1/*TC*, gives the apparent magnitude if the effect of time contraction is removed (*m_TC_*).



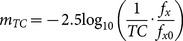
(13)
S1 Table presents the data for all SNe Ia in the SCP Union 2.1 compilation [53], [54] with the original values for redshift and distance modulus, as well as values that have been adjusted to compensate for the effects of time contraction.

In 1998 and 1999, two groups showed that SNe Ia with redshifts greater than 0.3–0.4 are dimmer than predicted from the linear application of the Hubble constant [56], [57]. This suggested that at earlier times in universe evolution, the rate of expansion was less than that of the Hubble constant. The shift from a slower rate of expansion to the current, faster Hubble constant rate provided evidence for an accelerating universe. In the Hubble diagram, SNe Ia at higher redshifts are located above the Hubble line (Fig. 1). Significantly, in the diagram adjusted for the effects of time contraction, the SNe Ia distribution straddles the Hubble line across all redshift values (Fig. 1).

Statistical analysis was performed to determine if the distribution of the TC-adjusted SNe Ia is consistent with a linear distribution. In agreement with previous reports [56], [57], the conventional Hubble SNe Ia distribution does not lie on a straight line (Wald–Wolfowitz Runs test, P<0.0001 using either weighted data that incorporates *m-M* errors from the SCP Union 2.1 compilation, or unweighted data; and analyzed with Prism 5 software by GraphPad Software). In contrast, the distribution of TC-adjusted SNe Ia does not statistically differ from the straight line derived from linear regression of the data set (Wald–Wolfowitz Runs test, P = 0.5507 with weighted data, and P = 0.1695 with unweighted data).

To further confirm that the TC-adjusted high-redshift SNe Ia are linear with low-redshift SNe Ia, the high-redshift SNe Ia were compared to a line derived from linear regression of low-redshift SNe Ia. The cut-off for low redshift SNe Ia was set to *z*<0.14 because this is the largest redshift value that contains the same number of SNe Ia in both data sets (194 of the 580 SNe Ia). Comparing the *z*<0.14 low-redshift Hubble line to the 100 highest-redshift SNe Ia using the Extra Sum-of-Squares F test shows that the distribution of the high-redshift SNe Ia in the conventional Hubble diagram is statistically different from the low-redshift Hubble line (P = 0.0004 with weighted data, and P = 0.0048 with unweighted data). In contrast, the distribution of the TC-adjusted high-redshift SNe Ia is not statistically different from the Hubble line (P = 0.4486 with weighted data, and P = 0.7863 with unweighted data). Therefore, adjusting the placement of SNe Ia to account for the effects of time contraction eliminates the statistical support for high-redshift SNe Ia that are dimmer than predicted from linear Hubble expansion.

### SNe Ia light curve durations are maintained in the time contraction scenario

SNe Ia have characteristic light curves that increase and decrease in intensity over a set time period. Cosmological time dilation alters the duration of the light curves that are observed on Earth by a factor of 1+*z* [58], [59]. The universal time dilation (UTD) scenario considered here implies that the duration of light curves for distant SNe Ia were time contracted at the time of emission when viewed from our current time scale. A central requirement for this scenario to be valid is that it must match the observed data; in this case, the duration of observed light curves for time-contracted SNe Ia must match the normally-observed duration. This requirement is met because while the duration of the light curve would have been compressed at the time of emission (relative to our time scale), there would be a correspondingly larger cosmological time dilation prior to the light being observed on Earth (as the light traversed from blueshift to redshift).

Changes in the light period correlate directly to changes in the duration of the light curve. To illustrate that the light periods of distant time-contracted SNe Ia have the normal periods upon observation, a specific SN Ia, sn2002fw, will be used as an example. As listed in S1 Table, sn2002fw has: *z* = 1.3; a time contraction ratio of *TC* = 1.367391; and a TC-adjusted redshift of *z_TC_* = 2.145. Under non-time-contraction conditions, the light period at the time of emission is *T_e_* = 1/*f_e_*_,_ where *f_e_* is the frequency of the light; and the period at observation is *T_o_* = *T_e_*(1+*z*) due to cosmological time dilation.

For this calculation, let *f_e_* = 1.

Under non-time-contraction conditions for SN 2002fw:



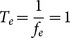





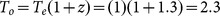



Time contraction increases the frequency of light at emission by the ratio *TC*, so that under time-contraction conditions for sn2002fw:



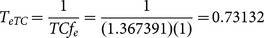





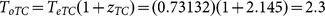



The observed light period is thus the same under both non-time-contracted conditions (*T_o_*) and time-contracted conditions (*T_oTC_*), and therefore both conditions will have the same observed light curve duration.

## Discussion

This study explores the potential validity of ALT, an alternate Lorentz transformation that is not widely known, and its implications for cosmology when integrated into the AST framework in which PRFs are linked to centers of gravitational mass. The failure to identify violations of Lorentz invariance has been used to support the widely-accepted SR theory. However, these experiments do not invalidate ALT, but rather act to restrict the localization of a potential PRF. Multiple experiments to test SR (analyzing light or subatomic particles moving at high relative speeds) have had the effect of restricting the localization of a putative PRF to the ECI. Complementary time dilation experiments that studied objects traveling at slower speeds for longer durations (e.g., airplanes and satellites) have provided evidence that the ECI acts as a PRF (broadly defined) to direct Lorentz/ALT transformations. Thus, the first class of experiments provides evidence that the only viable scenario for ALT is a PRF that is locally centered on the ECI, while the second class of experiments shows that the ECI does in fact act as a PRF for Lorentz/ALT transformations. Notably, GPS satellites traveling in inertial reference frames also experience directional time dilation relative to the ECI, and this finding is more compatible with AST than with SR.

The current situation, where there is a lack of compelling experimental evidence that distinguishes between SR and AST, allows one to countenance the possibility and implications of a valid AST. In the context of a valid AST, one can ask why the ECI functions as a PRF. The observation that objects moving in inertial reference frames experience directional time dilation relative to the ECI suggests that inertial reference frame status is not sufficient to confer PRF status. The most compelling hypothesis is that the ECI functions as a PRF because it is the local center of gravitational mass. This suggests that in an AST scenario, PRFs would not have fixed positions in the Universe, but would vary temporally and spatially based on the distribution of gravitational mass. New experimental data is required to definitively distinguish between SR and AST; and if the latter is supported, to inform theoretical models that describe how the effects of PRFs extend spatially and overlap.

The published interpretation of redshifts as kinematic recession velocities suggests that cosmological redshifts arise because cosmological objects in the present Universe move faster than objects in the past due to Hubble expansion. Combining this with ALT leads to a scenario of universal time dilation (UTD) in which the present Universe experiences time dilation relative to the past Universe. When viewed from our present (time-dilated) vantage point, cosmological objects in the past would have experienced time contraction that was associated with increased rates of light emissions and increased frequencies of emitted light. The UTD scenario would apply throughout the Universe, e.g., to observers in other PRFs or at rest with the CMB. The proposed universal nature of UTD is illustrated by equation (7), where the extent of time contraction is described in relation to the scale factor.

UTD has several implications, foremost of which is that the rate of time is not constant, and is linked to the rate of universe expansion. Because the effect of past time contraction includes the blueshifting of emissions (relative to our current time scale), light from distant cosmological objects would have undergone further changes in wavelength prior to reaching us (a greater redshift value); and therefore cosmological objects at high redshift would be older and more distant than currently envisioned.

Currently, the strongest and most direct evidence for an acceleration in the rate of universe expansion is that distant SNe Ia are less luminous than predicted by a linear regression of the Hubble constant [56], [57]. In the UTD scenario, SNe Ia that emitted light in the distant past would have experienced time contraction relative to our current time scale. To place time-contracted SNe Ia accurately on a Hubble-type diagram, the positions of the SNe Ia must be shifted to higher *z* values to reflect the increased change in *z* between the blueshifted emission and the observed redshift. Additionally, to compensate for the increased rate of light emissions of time-contracted SNe Ia, the SNe Ia must be shifted to higher distance modulus values to reflect the lower level of luminosity that would have resulted if the SNe Ia were emitting light at our current, slower, time-dilated rate. This latter adjustment is required so that all SNe Ia throughout the redshift spectrum have the same emission rate to allow them to function as standard candles with the same initial luminosity. Incorporating adjustments for the effects of time contraction produces a linear distribution of SNe Ia that has the effect of eliminating the signature of an accelerating universe. Given that the SNe Ia data is a direct readout of universe expansion [60], a linear distribution would have the effect of invalidating universe acceleration within the *z*<1.4 period.

Dark energy is proposed to drive the accelerated universe expansion, but its composition and mechanism of action are unknown. As stated in a review of dark energy: “… through most of the history of the universe dark matter or radiation dominated dark energy by many orders of magnitude. We happen to live at a time when dark energy has become important.”; “The universe has gone through three distinct eras: radiation dominated, *z*≥3000; matter dominated, 3000≥*z*≥0.5; and dark energy dominated, *z*≤0.5.”; and “… we expect that its effects at high redshift were very small, as otherwise it would have been difficult for large-scale structure to have formed…” [60]. The prevailing theory, while it can accurately model the effects of dark energy, is mechanistically not understood at multiple levels, including the nature of dark energy, and why it has significantly increased activity only in the most recent era. The UTD scenario is much simpler: universe expansion occurred at the Hubble constant through at least z<1.4 with no evidence for universe acceleration. In this scenario, the apparent non-linearity of high-redshift SNe on a Hubble diagram arises from a failure to incorporate the effects of time contraction, as only at higher redshifts are recession velocities large enough to produce appreciable time contraction effects.

Experimental support for a role of dark energy in universe acceleration comes from the analysis of four types of data: SNe Ia luminosity and redshift; the distribution of galaxy clusters; baryon acoustic oscillations; and the analysis of cosmic shear caused by gravitational lensing [60]. Of these, the SNe Ia data provides the most direct evidence for universe acceleration [60]. Notably, the signature of dark energy has only been observed with data for distant, high-redshift events. In contrast, the expected effect of dark energy on expansion within the solar system has not been observed [61]. This apparent contradiction does not apply to the UTD scenario, where the effects of time contraction manifest only at higher redshifts. Note that while the UTD scenario provides an alternate view of the recent increased effects of dark energy, it does not address the mechanistic basis for linear Hubble expansion, which may involve the cosmological constant/dark energy.

One argument against UTD is that it has the potential to disrupt current GRC theories, which are able to accurately model cosmological observations. In this regard, it should be noted that GRC theories have substantial inherent flexibilities that allow the theories to model diverse observations. The flexibility in these models derives from the ability to alter parameter values; and it is not unusual for these values to change in response to new experimental observations [62]. Historically, new GRC theories have been created when the prevailing theories were no longer able to accurately model new cosmological data, e.g., the creation of the ΛCDM model allowed the incorporation of the recently proposed expansion in the role of dark energy [63]. Presumably, if UTD is confirmed, it could be incorporated into future cosmological models.

In summary, current experimental evidence fails to definitively distinguish between SR and AST. This study shows that a valid AST would have significant implications for cosmology, including universal time dilation, increased ages and distances for high-redshift objects, and a linear, non-accelerating rate of universe expansion during the most recent era.

## Supporting Information

S1 Table **SNe Ia data with modifications for time contraction.**
(PDF)
