# Correction: Implications of an Absolute Simultaneity Theory for Cosmology and Universe Acceleration

**DOI:** 10.1371/journal.pone.0120187

**Published:** 2015-03-20

**Authors:** The PLOS ONE Staff

## Notice of Republication

This article was republished on February 3, 2015, to replace the missing Equation 5 in the online version and to correct errors in the legend of S1 Table in both the online and PDF versions. The publisher apologizes for the errors. The S1 Table legend has now been moved to within the file itself. Please download this article again to view the correct version. The originally published, uncorrected article and the republished, corrected article are provided here for reference.

## Supporting Information

S1 File Originally published, uncorrected article. (PDF)

S2 File Republished, corrected article. (PDF)
