# Supplementary material for: Implications of an Absolute Simultaneity Theory for Cosmology and Universe Acceleration
Source: PLoS One. 2014 Dec 23;9(12):e115550. doi: 10.1371/journal.pone.0115550 (PMC4275306; doi:10.1371/journal.pone.0115550)
Supplement: S1 Table — SNe Ia data with modifications for time contraction. (PDF) [file pone.0115550.s001.pdf]

Table S1: SNe Ia data with modifications for time contraction

Name	z	$m\text{-}M$	m	*flux*	TC	z_{TC}	m_{TC}	$m\text{-}M_{TC}$
1999aa	0.01500	34.164	14.855	1.143E-06	1.000111	0.01511	14.855	34.164
2006td	0.01500	34.380	15.072	9.362E-07	1.000111	0.01511	15.072	34.380
2007s	0.01500	34.111	14.803	1.199E-06	1.000111	0.01511	14.803	34.112
2005bo	0.01503	33.950	14.642	1.391E-06	1.000111	0.01514	14.642	33.950
2007ca	0.01510	34.526	15.218	8.183E-07	1.000112	0.01521	15.218	34.526
1994s	0.01517	34.102	14.794	1.209E-06	1.000113	0.01528	14.794	34.102
2001bf	0.01520	34.017	14.709	1.308E-06	1.000114	0.01532	14.709	34.017
2002do	0.01520	34.258	14.950	1.047E-06	1.000114	0.01532	14.950	34.258
2006cm	0.01530	34.707	15.399	6.924E-07	1.000115	0.01542	15.399	34.707
2001cn	0.01544	33.941	14.633	1.402E-06	1.000117	0.01556	14.633	33.941
2001da	0.01600	34.174	14.866	1.132E-06	1.000126	0.01613	14.866	34.174
2001v	0.01600	33.825	14.516	1.561E-06	1.000126	0.01613	14.517	33.825
2002hw	0.01630	34.453	15.145	8.749E-07	1.000131	0.01643	15.145	34.453
1996bo	0.01632	34.017	14.709	1.307E-06	1.000131	0.01645	14.709	34.018
2001cz	0.01635	34.044	14.736	1.275E-06	1.000131	0.01648	14.736	34.044
2000dk	0.01645	34.181	14.873	1.124E-06	1.000133	0.01659	14.873	34.181
1997y	0.01656	34.344	15.036	9.677E-07	1.000135	0.01670	15.036	34.344
1996bv	0.01673	34.226	14.918	1.078E-06	1.000138	0.01687	14.918	34.226
1998ef	0.01674	34.003	14.695	1.325E-06	1.000138	0.01688	14.695	34.003
1998co	0.01699	34.379	15.071	9.370E-07	1.000142	0.01714	15.071	34.379
1998v	0.01717	34.261	14.953	1.045E-06	1.000145	0.01732	14.953	34.261
1992bo	0.01723	34.654	15.346	7.270E-07	1.000146	0.01738	15.346	34.655

2001g	0.01730	34.250	14.942	1.055E-06	1.000147	0.01745	14.942	34.250
2006le	0.01730	34.230	14.922	1.075E-06	1.000147	0.01745	14.922	34.230
1999ek	0.01761	34.344	15.036	9.677E-07	1.000152	0.01776	15.036	34.344
2006ax	0.01793	34.350	15.042	9.624E-07	1.000158	0.01809	15.042	34.350
2005a	0.01832	34.372	15.064	9.431E-07	1.000165	0.01848	15.064	34.372
2002jy	0.01870	35.048	15.740	5.058E-07	1.000172	0.01887	15.740	35.048
2008l	0.01890	34.375	15.067	9.405E-07	1.000175	0.01908	15.067	34.375
2006ej	0.01920	34.737	15.429	6.739E-07	1.000181	0.01938	15.429	34.737
2007ci	0.01920	34.492	15.183	8.446E-07	1.000181	0.01938	15.184	34.492
1999gd	0.01926	34.953	15.644	5.524E-07	1.000182	0.01945	15.645	34.953
2002kf	0.01950	34.757	15.449	6.615E-07	1.000186	0.01969	15.449	34.757
1992bc	0.01960	34.585	15.277	7.748E-07	1.000188	0.01979	15.277	34.585
2005ki	0.02037	34.656	15.348	7.256E-07	1.000203	0.02058	15.349	34.657
2005ls	0.02050	34.617	15.309	7.522E-07	1.000206	0.02071	15.309	34.618
2006kf	0.02080	34.795	15.487	6.384E-07	1.000212	0.02102	15.488	34.796
2007au	0.02090	34.703	15.395	6.952E-07	1.000214	0.02112	15.395	34.703
2003w	0.02110	34.661	15.353	7.227E-07	1.000218	0.02132	15.353	34.661
2006et	0.02120	34.847	15.539	6.087E-07	1.000220	0.02142	15.539	34.847
2006bq	0.02150	34.933	15.625	5.626E-07	1.000226	0.02173	15.625	34.933
2000fa	0.02179	34.974	15.666	5.417E-07	1.000232	0.02203	15.666	34.974
2007bc	0.02190	34.849	15.541	6.077E-07	1.000235	0.02214	15.541	34.849
1995ak	0.02198	34.853	15.545	6.054E-07	1.000236	0.02222	15.545	34.853
2001n	0.02210	34.912	15.603	5.736E-07	1.000239	0.02234	15.604	34.912
2004bg	0.02210	34.922	15.614	5.681E-07	1.000239	0.02234	15.614	34.922

2001cp	0.02240	34.947	15.639	5.552E-07	1.000245	0.02265	15.639	34.947
2006ar	0.02290	35.197	15.889	4.411E-07	1.000256	0.02316	15.889	35.197
2007qe	0.02290	35.139	15.830	4.654E-07	1.000256	0.02316	15.831	35.139
2005m	0.02297	35.134	15.826	4.674E-07	1.000258	0.02324	15.826	35.134
2006sr	0.02300	35.071	15.762	4.955E-07	1.000259	0.02326	15.763	35.071
2000cn	0.02321	35.086	15.777	4.887E-07	1.000263	0.02348	15.778	35.086
2006cp	0.02330	34.882	15.574	5.894E-07	1.000265	0.02357	15.574	34.882
2006mp	0.02330	35.198	15.890	4.405E-07	1.000265	0.02357	15.890	35.199
1998eg	0.02354	35.170	15.861	4.523E-07	1.000271	0.02381	15.862	35.170
2006ac	0.02390	35.035	15.727	5.120E-07	1.000279	0.02419	15.727	35.035
2000bh	0.02395	34.969	15.661	5.442E-07	1.000280	0.02424	15.661	34.969
2003it	0.02400	35.176	15.868	4.496E-07	1.000281	0.02429	15.868	35.176
2005bg	0.02419	35.052	15.744	5.039E-07	1.000286	0.02448	15.744	35.053
2007f	0.02420	35.189	15.881	4.441E-07	1.000286	0.02449	15.882	35.190
1994m	0.02431	35.109	15.801	4.780E-07	1.000289	0.02461	15.802	35.110
2000ca	0.02453	35.058	15.750	5.012E-07	1.000294	0.02483	15.750	35.058
2007cq	0.02470	34.914	15.606	5.725E-07	1.000298	0.02501	15.606	34.914
2002he	0.02480	35.256	15.948	4.176E-07	1.000300	0.02511	15.948	35.257
2002bf	0.02490	34.804	15.496	6.335E-07	1.000302	0.02521	15.496	34.804
2008bf	0.02510	34.948	15.640	5.546E-07	1.000307	0.02542	15.640	34.949
2006br	0.02550	35.720	16.412	2.724E-07	1.000317	0.02583	16.412	35.721
2003ch	0.02560	35.685	16.377	2.814E-07	1.000320	0.02593	16.377	35.685
2005ms	0.02590	35.416	16.108	3.605E-07	1.000327	0.02624	16.108	35.416
2005mc	0.02600	35.381	16.073	3.722E-07	1.000329	0.02634	16.073	35.382

1999gp	0.02604	35.367	16.059	3.770E-07	1.000330	0.02638	16.059	35.368
2003u	0.02610	35.360	16.052	3.794E-07	1.000332	0.02644	16.053	35.361
1992p	0.02649	35.481	16.173	3.396E-07	1.000342	0.02684	16.173	35.481
2007co	0.02660	35.323	16.015	3.925E-07	1.000345	0.02695	16.016	35.324
2005na	0.02681	35.260	15.952	4.161E-07	1.000350	0.02717	15.952	35.261
1992ag	0.02734	35.086	15.778	4.886E-07	1.000364	0.02772	15.778	35.086
1996c	0.02750	35.650	16.342	2.907E-07	1.000368	0.02788	16.342	35.650
2004gs	0.02757	35.475	16.167	3.413E-07	1.000370	0.02795	16.168	35.476
2006gj	0.02770	35.705	16.397	2.762E-07	1.000373	0.02808	16.397	35.705
1998ab	0.02787	35.093	15.785	4.852E-07	1.000378	0.02825	15.786	35.094
2002de	0.02830	35.520	16.212	3.275E-07	1.000389	0.02870	16.213	35.521
2005eq	0.02840	35.548	16.240	3.192E-07	1.000392	0.02880	16.240	35.549
1993ah	0.02849	35.347	16.038	3.843E-07	1.000395	0.02889	16.039	35.347
2002hu	0.02920	35.993	16.684	2.120E-07	1.000414	0.02963	16.685	35.993
2004ef	0.02980	35.471	16.163	3.427E-07	1.000431	0.03025	16.163	35.471
1997dg	0.02996	35.972	16.664	2.160E-07	1.000436	0.03040	16.665	35.973
2002ck	0.03030	35.628	16.320	2.965E-07	1.000446	0.03076	16.321	35.629
2001ba	0.03053	35.599	16.291	3.045E-07	1.000452	0.03100	16.292	35.600
1990o	0.03060	35.550	16.242	3.185E-07	1.000454	0.03107	16.243	35.551
2006bw	0.03080	35.629	16.321	2.963E-07	1.000460	0.03127	16.321	35.629
2006en	0.03080	35.779	16.471	2.580E-07	1.000460	0.03127	16.471	35.780
2006qo	0.03080	35.594	16.285	3.061E-07	1.000460	0.03127	16.286	35.594
2001ay	0.03090	35.929	16.621	2.246E-07	1.000463	0.03138	16.622	35.930
2001ie	0.03120	35.627	16.319	2.969E-07	1.000472	0.03169	16.319	35.627

2007r	0.03120	35.883	16.575	2.345E-07	1.000472	0.03169	16.575	35.883
2006az	0.03150	35.651	16.343	2.903E-07	1.000481	0.03200	16.344	35.652
1999cc	0.03153	35.729	16.421	2.702E-07	1.000482	0.03203	16.421	35.729
2007ai	0.03200	35.827	16.519	2.469E-07	1.000496	0.03251	16.519	35.827
2007bd	0.03200	35.588	16.280	3.075E-07	1.000496	0.03251	16.281	35.589
2004as	0.03210	35.896	16.588	2.317E-07	1.000499	0.03262	16.588	35.897
2006os	0.03210	35.648	16.340	2.911E-07	1.000499	0.03262	16.340	35.649
2006te	0.03210	35.870	16.562	2.372E-07	1.000499	0.03262	16.563	35.871
2004gc	0.03213	35.378	16.070	3.733E-07	1.000500	0.03265	16.071	35.379
2006bt	0.03250	35.813	16.505	2.500E-07	1.000512	0.03303	16.505	35.814
2006cc	0.03270	36.094	16.786	1.930E-07	1.000518	0.03323	16.787	36.095
2006s	0.03290	35.942	16.634	2.221E-07	1.000524	0.03344	16.634	35.942
2005iq	0.03291	35.968	16.660	2.168E-07	1.000524	0.03345	16.660	35.968
2004l	0.03340	35.880	16.572	2.351E-07	1.000540	0.03396	16.572	35.881
2006gr	0.03350	35.974	16.666	2.156E-07	1.000543	0.03406	16.666	35.974
2003iv	0.03360	36.005	16.697	2.095E-07	1.000546	0.03416	16.698	36.006
2003cq	0.03370	35.844	16.536	2.431E-07	1.000549	0.03427	16.536	35.844
2003kc	0.03410	35.842	16.534	2.435E-07	1.000562	0.03468	16.534	35.843
2005eu	0.03410	35.943	16.634	2.219E-07	1.000562	0.03468	16.635	35.943
2008af	0.03410	35.770	16.462	2.601E-07	1.000562	0.03468	16.463	35.771
2002g	0.03450	35.978	16.670	2.148E-07	1.000575	0.03510	16.671	35.979
1994t	0.03572	35.961	16.652	2.183E-07	1.000616	0.03636	16.653	35.961
1996bl	0.03600	35.821	16.513	2.482E-07	1.000625	0.03665	16.514	35.822
2002hd	0.03600	35.679	16.371	2.829E-07	1.000625	0.03665	16.372	35.680

2006mo	0.03600	36.146	16.838	1.840E-07	1.000625	0.03665	16.839	36.147
2001eh	0.03620	35.987	16.679	2.131E-07	1.000632	0.03686	16.679	35.988
2000cf	0.03646	36.134	16.826	1.860E-07	1.000641	0.03712	16.827	36.135
1992bg	0.03648	35.905	16.597	2.297E-07	1.000642	0.03715	16.598	35.906
2007o	0.03660	35.971	16.663	2.161E-07	1.000646	0.03727	16.664	35.972
2007cp	0.03770	35.795	16.487	2.542E-07	1.000685	0.03841	16.488	35.796
1999aw	0.03930	36.334	17.026	1.547E-07	1.000743	0.04007	17.027	36.335
2005lz	0.04020	36.375	17.066	1.491E-07	1.000777	0.04101	17.067	36.375
2001az	0.04060	36.366	17.057	1.503E-07	1.000792	0.04142	17.058	36.366
2005hf	0.04210	36.400	17.092	1.456E-07	1.000850	0.04299	17.093	36.401
1992bl	0.04223	36.336	17.028	1.544E-07	1.000856	0.04312	17.029	36.337
2006cf	0.04230	36.393	17.085	1.466E-07	1.000858	0.04319	17.086	36.394
2006cz	0.04250	35.928	16.620	2.249E-07	1.000866	0.04340	16.621	35.929
2005ku	0.04372	36.387	17.078	1.474E-07	1.000916	0.04467	17.079	36.388
2005hc	0.04498	36.550	17.242	1.268E-07	1.000968	0.04599	17.243	36.551
1992bh	0.04530	36.640	17.332	1.167E-07	1.000981	0.04632	17.333	36.641
2004gu	0.04697	36.494	17.186	1.335E-07	1.001053	0.04807	17.187	36.496
2006eq	0.04839	36.686	17.378	1.119E-07	1.001117	0.04956	17.379	36.687
1995ac	0.04882	36.382	17.074	1.481E-07	1.001136	0.05001	17.075	36.383
1993ac	0.04895	36.732	17.423	1.073E-07	1.001142	0.05015	17.425	36.733
2006cq	0.04910	36.730	17.422	1.075E-07	1.001149	0.05031	17.423	36.731
1990af	0.04992	36.567	17.259	1.249E-07	1.001187	0.05117	17.260	36.568
1993ag	0.05004	36.682	17.374	1.123E-07	1.001192	0.05130	17.376	36.684
2006ot	0.05220	36.674	17.366	1.131E-07	1.001295	0.05356	17.368	36.676

1993o	0.05293	36.818	17.510	9.913E-08	1.001330	0.05433	17.511	36.819
1998dx	0.05371	36.476	17.168	1.357E-07	1.001369	0.05515	17.170	36.478
1999ao	0.05440	36.954	17.646	8.740E-08	1.001403	0.05588	17.648	36.956
2003ic	0.05460	36.610	17.301	1.201E-07	1.001413	0.05609	17.303	36.611
2006py	0.05668	36.964	17.655	8.666E-08	1.001520	0.05829	17.657	36.965
2005hj	0.05760	37.080	17.772	7.783E-08	1.001569	0.05926	17.774	37.082
2001ah	0.05830	37.033	17.724	8.132E-08	1.001606	0.06000	17.726	37.034
2006ob	0.05830	37.058	17.750	7.942E-08	1.001606	0.06000	17.752	37.060
2006oa	0.05890	37.112	17.803	7.561E-08	1.001638	0.06063	17.805	37.113
2005ho	0.06184	37.131	17.823	7.428E-08	1.001800	0.06375	17.825	37.133
1992bs	0.06267	37.483	18.175	5.369E-08	1.001848	0.06463	18.177	37.485
2005kt	0.06386	37.316	18.008	6.264E-08	1.001917	0.06590	18.010	37.318
2007ae	0.06430	37.176	17.867	7.130E-08	1.001942	0.06637	17.869	37.178
2006an	0.06510	37.307	17.999	6.318E-08	1.001989	0.06722	18.001	37.309
2005if	0.06644	37.372	18.064	5.949E-08	1.002070	0.06865	18.066	37.374
2006cj	0.06840	37.731	18.423	4.273E-08	1.002190	0.07074	18.425	37.734
2006on	0.06880	37.487	18.178	5.353E-08	1.002214	0.07117	18.181	37.489
2006al	0.06900	37.566	18.258	4.976E-08	1.002227	0.07138	18.260	37.568
1993b	0.07009	37.447	18.139	5.554E-08	1.002295	0.07254	18.141	37.449
1992ae	0.07461	37.588	18.280	4.875E-08	1.002590	0.07739	18.283	37.591
2005ir	0.07535	37.580	18.272	4.912E-08	1.002640	0.07819	18.275	37.583
1999bp	0.07840	37.682	18.374	4.471E-08	1.002850	0.08147	18.377	37.685
1992bp	0.07858	37.488	18.180	5.346E-08	1.002862	0.08166	18.183	37.491
2005ag	0.08005	37.686	18.378	4.456E-08	1.002966	0.08325	18.381	37.689

2006bu	0.08430	38.052	18.744	3.181E-08	1.003277	0.08785	18.747	38.055
2005ff	0.08569	37.997	18.689	3.344E-08	1.003382	0.08936	18.693	38.001
2005ed	0.08570	37.994	18.686	3.355E-08	1.003382	0.08937	18.689	37.997
2005gb	0.08585	37.952	18.644	3.486E-08	1.003394	0.08954	18.648	37.956
1992br	0.08759	38.229	18.921	2.702E-08	1.003527	0.09142	18.925	38.233
2005iu	0.08902	37.828	18.520	3.907E-08	1.003638	0.09298	18.524	37.832
2005ex	0.09294	38.146	18.838	2.917E-08	1.003951	0.09726	18.842	38.150
2005je	0.09315	38.281	18.973	2.576E-08	1.003969	0.09749	18.977	38.285
2005fn	0.09391	38.173	18.865	2.845E-08	1.004031	0.09832	18.869	38.177
1992aq	0.10092	38.457	19.149	2.191E-08	1.004625	0.10601	19.154	38.462
2005lk	0.10272	38.487	19.179	2.130E-08	1.004784	0.10799	19.184	38.492
2005hn	0.10671	38.637	19.329	1.856E-08	1.005145	0.11241	19.334	38.642
2005jh	0.10864	38.651	19.342	1.833E-08	1.005323	0.11454	19.348	38.656
2005ml	0.11304	38.551	19.243	2.008E-08	1.005740	0.11943	19.250	38.558
2005kp	0.11471	38.669	19.361	1.801E-08	1.005902	0.12129	19.367	38.675
2005hr	0.11635	38.746	19.438	1.678E-08	1.006063	0.12312	19.445	38.753
2005fv	0.11728	38.746	19.438	1.678E-08	1.006155	0.12415	19.444	38.753
2005fh	0.11763	38.579	19.271	1.957E-08	1.006190	0.12454	19.278	38.586
2005hx	0.11967	38.756	19.447	1.664E-08	1.006395	0.12683	19.454	38.762
2005fz	0.12283	38.800	19.492	1.597E-08	1.006718	0.13037	19.499	38.807
1999bi	0.12410	38.822	19.514	1.565E-08	1.006850	0.13180	19.521	38.829
2005ij	0.12427	38.720	19.412	1.719E-08	1.006868	0.13200	19.419	38.727
1996ab	0.12440	39.045	19.737	1.275E-08	1.006882	0.13214	19.744	39.052
2005gp	0.12647	38.710	19.402	1.734E-08	1.007100	0.13447	19.410	38.718

2005ei	0.12669	38.915	19.607	1.436E-08	1.007123	0.13471	19.615	38.923
2005hz	0.12873	38.864	19.556	1.505E-08	1.007340	0.13701	19.564	38.872
2005ez	0.12928	38.918	19.610	1.432E-08	1.007400	0.13763	19.618	38.926
1999bn	0.12990	38.979	19.671	1.354E-08	1.007467	0.13834	19.679	38.987
2005fj	0.14179	39.274	19.966	1.032E-08	1.008804	0.15184	19.975	39.283
2005fw	0.14240	39.116	19.808	1.193E-08	1.008876	0.15254	19.818	39.126
2005ld	0.14371	39.186	19.878	1.119E-08	1.009028	0.15403	19.887	39.196
1999bm	0.14410	38.836	19.528	1.545E-08	1.009075	0.15448	19.538	38.846
2005gx	0.14462	39.291	19.983	1.016E-08	1.009136	0.15508	19.993	39.301
2005ln	0.14567	39.057	19.749	1.261E-08	1.009261	0.15628	19.759	39.067
10106	0.14629	39.559	20.251	7.937E-09	1.009335	0.15699	20.261	39.569
2005ey	0.14703	39.304	19.995	1.004E-08	1.009423	0.15783	20.006	39.314
2005fm	0.15186	39.156	19.848	1.151E-08	1.010010	0.16339	19.858	39.167
2005hy	0.15463	39.324	20.016	9.856E-09	1.010354	0.16659	20.027	39.335
4064	0.15525	39.317	20.009	9.916E-09	1.010431	0.16730	20.020	39.329
1999ar	0.15610	39.229	19.921	1.075E-08	1.010539	0.16828	19.932	39.241
e020	0.15900	39.416	20.108	9.051E-09	1.010906	0.17164	20.120	39.428
2005gd	0.15989	39.351	20.042	9.617E-09	1.011020	0.17267	20.054	39.362
2005fa	0.16086	39.319	20.011	9.899E-09	1.011145	0.17380	20.023	39.331
2005gc	0.16380	39.395	20.086	9.235E-09	1.011527	0.17721	20.099	39.407
2005is	0.17063	39.475	20.167	8.576E-09	1.012435	0.18519	20.180	39.488
1997i	0.17200	39.302	19.994	1.005E-08	1.012621	0.18679	20.008	39.316
2005ga	0.17274	39.503	20.195	8.358E-09	1.012722	0.18766	20.209	39.517
2005hp	0.17391	39.532	20.224	8.137E-09	1.012882	0.18903	20.238	39.546

2005hv	0.17760	40.071	20.763	4.954E-09	1.013392	0.19337	20.777	40.085
1999dr	0.17800	39.455	20.147	8.737E-09	1.013448	0.19384	20.161	39.469
2005jl	0.17969	39.721	20.413	6.837E-09	1.013685	0.19583	20.428	39.736
1997n	0.18000	40.190	20.881	4.441E-09	1.013729	0.19620	20.896	40.204
2005ft	0.18012	39.639	20.330	7.376E-09	1.013746	0.19634	20.345	39.653
k429	0.18100	39.683	20.375	7.081E-09	1.013870	0.19738	20.390	39.698
2005gj	0.18222	39.594	20.286	7.687E-09	1.014043	0.19882	20.301	39.609
2005id	0.18255	39.784	20.476	6.451E-09	1.014090	0.19921	20.491	39.799
2005ko	0.18357	39.711	20.403	6.902E-09	1.014235	0.20042	20.418	39.726
2005ht	0.18581	39.824	20.516	6.218E-09	1.014558	0.20308	20.532	39.840
1999dv	0.18600	39.712	20.404	6.893E-09	1.014585	0.20330	20.420	39.728
2005jk	0.18885	39.792	20.484	6.404E-09	1.015000	0.20669	20.500	39.808
2005eg	0.18971	39.940	20.631	5.590E-09	1.015125	0.20770	20.648	39.956
2005fu	0.19215	40.039	20.731	5.100E-09	1.015485	0.21061	20.748	40.056
2005fy	0.19432	39.962	20.653	5.478E-09	1.015808	0.21320	20.670	39.979
2005kn	0.19672	39.924	20.616	5.669E-09	1.016168	0.21606	20.634	39.942
2005iz	0.20061	39.852	20.544	6.058E-09	1.016760	0.22073	20.562	39.870
2005jm	0.20261	39.968	20.660	5.447E-09	1.017067	0.22313	20.678	39.986
2005ge	0.20498	40.025	20.716	5.170E-09	1.017435	0.22599	20.735	40.043
d086	0.20500	39.904	20.596	5.778E-09	1.017438	0.22601	20.614	39.923
2005jp	0.21094	39.984	20.676	5.366E-09	1.018372	0.23319	20.696	40.004
2005fp	0.21159	40.560	21.252	3.156E-09	1.018475	0.23397	21.272	40.580
2005jc	0.21163	39.980	20.672	5.385E-09	1.018482	0.23402	20.692	40.000
2005mi	0.21255	40.088	20.779	4.878E-09	1.018629	0.23514	20.799	40.108

h363	0.21300	40.109	20.801	4.782E-09	1.018701	0.23568	20.821	40.129
2005ji	0.21457	40.202	20.894	4.391E-09	1.018953	0.23759	20.914	40.222
1999dy	0.21500	40.378	21.070	3.733E-09	1.019023	0.23811	21.090	40.399
2005fe	0.21554	40.297	20.988	4.024E-09	1.019110	0.23877	21.009	40.317
2002kc	0.21600	40.556	21.248	3.168E-09	1.019184	0.23933	21.269	40.577
n404	0.21600	40.405	21.097	3.642E-09	1.019184	0.23933	21.117	40.426
2005lh	0.21658	40.360	21.052	3.794E-09	1.019279	0.24004	21.073	40.381
g005	0.21800	40.075	20.767	4.933E-09	1.019509	0.24176	20.788	40.096
2005jo	0.21835	40.221	20.913	4.314E-09	1.019566	0.24219	20.934	40.242
2005hu	0.21859	40.199	20.891	4.402E-09	1.019604	0.24247	20.912	40.220
2005gg	0.22853	40.254	20.946	4.183E-09	1.021255	0.25464	20.969	40.277
2005fl	0.23278	40.205	20.896	4.380E-09	1.021978	0.25987	20.920	40.228
e132	0.23900	40.259	20.951	4.165E-09	1.023051	0.26756	20.976	40.284
1995ao	0.24000	40.742	21.434	2.669E-09	1.023226	0.26880	21.459	40.767
2005ll	0.24250	40.130	20.822	4.689E-09	1.023665	0.27191	20.848	40.156
2005gr	0.24438	40.202	20.894	4.390E-09	1.023996	0.27424	20.920	40.228
2005gf	0.24851	40.272	20.964	4.115E-09	1.024732	0.27939	20.991	40.299
03D3bh	0.24860	40.611	21.303	3.012E-09	1.024749	0.27950	21.330	40.638
2005gs	0.24951	40.781	21.473	2.576E-09	1.024912	0.28064	21.499	40.808
2005ia	0.25067	40.746	21.437	2.661E-09	1.025120	0.28208	21.464	40.773
2005jz	0.25174	40.542	21.234	3.211E-09	1.025314	0.28343	21.261	40.569
2005le	0.25249	40.518	21.210	3.280E-09	1.025449	0.28436	21.238	40.546
2005li	0.25549	40.486	21.178	3.380E-09	1.025996	0.28813	21.206	40.514
2005jb	0.25648	40.611	21.303	3.012E-09	1.026176	0.28937	21.331	40.639

2005ih	0.25750	40.594	21.286	3.059E-09	1.026364	0.29065	21.314	40.622
2005gh	0.25774	40.651	21.343	2.902E-09	1.026408	0.29096	21.372	40.680
2005ju	0.25803	40.645	21.337	2.920E-09	1.026461	0.29132	21.365	40.673
1999du	0.26000	40.823	21.515	2.477E-09	1.026825	0.29380	21.544	40.852
2005fo	0.26053	40.808	21.500	2.512E-09	1.026924	0.29447	21.529	40.837
2005fd	0.26059	40.523	21.215	3.266E-09	1.026934	0.29454	21.244	40.552
04D3ez	0.26300	40.635	21.327	2.947E-09	1.027383	0.29758	21.356	40.664
2005fi	0.26349	40.767	21.459	2.608E-09	1.027474	0.29820	21.489	40.797
2005go	0.26365	40.548	21.240	3.191E-09	1.027504	0.29840	21.269	40.578
2005ix	0.26576	40.498	21.190	3.342E-09	1.027900	0.30108	21.220	40.528
m043	0.26600	40.391	21.083	3.690E-09	1.027945	0.30138	21.112	40.421
1999dx	0.26900	40.776	21.468	2.587E-09	1.028511	0.30518	21.499	40.807
2005jy	0.27043	40.669	21.361	2.855E-09	1.028783	0.30700	21.392	40.700
k396	0.27100	40.532	21.224	3.238E-09	1.028891	0.30772	21.255	40.563
2005mo	0.27345	40.727	21.419	2.706E-09	1.029360	0.31084	21.450	40.759
k425	0.27400	40.725	21.416	2.713E-09	1.029465	0.31154	21.448	40.756
2005gw	0.27544	40.743	21.435	2.666E-09	1.029742	0.31337	21.467	40.775
2005gt	0.27785	40.838	21.530	2.444E-09	1.030208	0.31645	21.562	40.870
1999fw	0.27800	40.566	21.258	3.139E-09	1.030236	0.31664	21.290	40.599
2005ie	0.27892	40.768	21.460	2.607E-09	1.030416	0.31782	21.492	40.800
2005ig	0.27945	40.501	21.193	3.332E-09	1.030519	0.31850	21.226	40.534
p455	0.28400	40.835	21.527	2.450E-09	1.031408	0.32433	21.561	40.869
03D4ag	0.28500	40.854	21.546	2.407E-09	1.031605	0.32561	21.580	40.888
m027	0.28600	41.211	21.903	1.733E-09	1.031802	0.32690	21.937	41.245

2005fr	0.28662	41.038	21.730	2.032E-09	1.031925	0.32769	21.764	41.073
2005fx	0.28842	40.843	21.535	2.433E-09	1.032282	0.33001	21.569	40.877
03D3ba	0.29120	40.839	21.531	2.441E-09	1.032837	0.33360	21.566	40.874
2005ii	0.29247	40.919	21.611	2.268E-09	1.033091	0.33524	21.646	40.954
2005fc	0.29559	41.137	21.829	1.855E-09	1.033719	0.33927	21.865	41.173
2005lo	0.29752	40.772	21.464	2.596E-09	1.034110	0.34178	21.500	40.809
2005lf	0.29841	41.062	21.754	1.987E-09	1.034291	0.34293	21.791	41.099
2005iv	0.29878	41.029	21.721	2.049E-09	1.034366	0.34341	21.758	41.066
1996j	0.30000	40.963	21.655	2.178E-09	1.034615	0.34500	21.692	41.000
2005hs	0.30031	40.847	21.539	2.424E-09	1.034679	0.34541	21.576	40.884
2005lp	0.30176	41.494	22.186	1.336E-09	1.034974	0.34728	22.223	41.531
g055	0.30200	41.311	22.003	1.581E-09	1.035025	0.34760	22.040	41.348
2005jg	0.30240	41.033	21.725	2.042E-09	1.035107	0.34812	21.763	41.071
2005it	0.30858	40.908	21.599	2.292E-09	1.036384	0.35619	21.638	40.946
d117	0.30900	41.174	21.866	1.794E-09	1.036471	0.35674	21.905	41.213
n278	0.30900	40.861	21.553	2.393E-09	1.036471	0.35674	21.592	40.900
2005ic	0.30949	41.018	21.710	2.070E-09	1.036574	0.35739	21.749	41.057
2005ik	0.30955	41.193	21.884	1.763E-09	1.036585	0.35746	21.923	41.232
2005jd	0.31288	41.014	21.706	2.077E-09	1.037283	0.36183	21.746	41.054
m062	0.31400	41.232	21.924	1.700E-09	1.037518	0.36330	21.964	41.272
2005ka	0.31643	41.395	22.087	1.463E-09	1.038030	0.36649	22.127	41.436
1997ac	0.32000	41.245	21.936	1.680E-09	1.038788	0.37120	21.978	41.286
2005jn	0.32045	41.103	21.795	1.915E-09	1.038883	0.37179	21.836	41.144
2005ja	0.32640	40.972	21.664	2.159E-09	1.040159	0.37966	21.707	41.015

b016	0.32900	41.340	22.032	1.539E-09	1.040723	0.38312	22.075	41.384
2005gu	0.33051	41.228	21.920	1.706E-09	1.041051	0.38513	21.963	41.272
2005gy	0.33063	41.052	21.744	2.006E-09	1.041078	0.38529	21.788	41.096
03D1fc	0.33100	41.078	21.770	1.958E-09	1.041157	0.38578	21.814	41.122
e029	0.33200	41.255	21.947	1.664E-09	1.041375	0.38711	21.991	41.299
04D3kr	0.33730	41.294	21.986	1.605E-09	1.042538	0.39419	22.032	41.340
2005fs	0.33880	41.305	21.997	1.589E-09	1.042869	0.39620	22.043	41.351
2001iw	0.33960	41.084	21.776	1.948E-09	1.043046	0.39726	21.822	41.130
d087	0.34000	41.213	21.905	1.730E-09	1.043134	0.39780	21.951	41.259
g097	0.34000	41.080	21.772	1.955E-09	1.043134	0.39780	21.818	41.126
04D3nh	0.34020	41.328	22.020	1.556E-09	1.043179	0.39807	22.066	41.374
m193	0.34100	40.992	21.683	2.122E-09	1.043356	0.39914	21.729	41.038
d149	0.34200	41.385	22.076	1.477E-09	1.043578	0.40048	22.123	41.431
h364	0.34400	41.173	21.865	1.795E-09	1.044024	0.40317	21.911	41.220
03D1bp	0.34600	41.365	22.056	1.505E-09	1.044471	0.40586	22.104	41.412
h359	0.34800	41.590	22.281	1.223E-09	1.044920	0.40855	22.329	41.637
2005mq	0.34835	41.308	22.000	1.585E-09	1.044997	0.40902	22.047	41.356
2005lg	0.34858	41.301	21.993	1.595E-09	1.045051	0.40934	22.041	41.349
e136	0.35200	41.424	22.116	1.425E-09	1.045822	0.41395	22.164	41.472
1998as	0.35500	41.342	22.034	1.536E-09	1.046504	0.41801	22.084	41.392
04D2fs	0.35700	41.426	22.117	1.422E-09	1.046960	0.42072	22.167	41.475
1688	0.35751	41.329	22.021	1.555E-09	1.047076	0.42141	22.071	41.379
04D3fk	0.35780	41.435	22.127	1.410E-09	1.047143	0.42181	22.177	41.485
2005jt	0.36003	41.157	21.849	1.821E-09	1.047655	0.42485	21.900	41.208

2005gv	0.36193	41.384	22.076	1.477E-09	1.048092	0.42743	22.127	41.435
d093	0.36300	41.552	22.244	1.266E-09	1.048338	0.42888	22.295	41.603
2005jj	0.36660	41.860	22.552	9.535E-10	1.049172	0.43380	22.604	41.912
n263	0.36800	41.468	22.160	1.368E-09	1.049497	0.43571	22.212	41.520
04D2cf	0.36900	41.634	22.326	1.174E-09	1.049730	0.43708	22.379	41.687
03D3ay	0.37090	41.669	22.361	1.137E-09	1.050174	0.43968	22.414	41.722
1997o	0.37400	43.185	23.877	2.813E-10	1.050901	0.44394	23.931	43.239
2005lq	0.37897	41.581	22.273	1.232E-09	1.052073	0.45077	22.328	41.637
2005jw	0.37966	41.433	22.125	1.413E-09	1.052239	0.45173	22.180	41.488
1996k	0.38000	42.063	22.755	7.907E-10	1.052319	0.45220	22.810	42.119
2005mm	0.38036	41.657	22.349	1.149E-09	1.052404	0.45270	22.405	41.713
1166	0.38042	41.272	21.964	1.638E-09	1.052418	0.45277	22.020	41.328
g052	0.38300	41.642	22.334	1.165E-09	1.053033	0.45634	22.390	41.698
2005kq	0.38730	41.892	22.584	9.253E-10	1.054062	0.46230	22.641	41.950
1995ba	0.38800	42.208	22.899	6.922E-10	1.054231	0.46327	22.957	42.265
2005gq	0.38929	41.603	22.294	1.208E-09	1.054541	0.46506	22.352	41.660
5737	0.39160	41.509	22.201	1.318E-09	1.055098	0.46827	22.259	41.567
2005mh	0.39397	41.781	22.473	1.025E-09	1.055674	0.47158	22.532	41.840
2001iv	0.39650	41.488	22.180	1.343E-09	1.056288	0.47511	22.239	41.547
g142	0.39900	41.488	22.180	1.343E-09	1.056898	0.47860	22.240	41.548
2005hq	0.39960	41.729	22.421	1.076E-09	1.057045	0.47944	22.481	41.789
1995aw	0.40000	42.313	23.005	6.283E-10	1.057143	0.48000	23.065	42.373
d085	0.40100	41.695	22.387	1.109E-09	1.057388	0.48140	22.448	41.756
f308	0.40100	42.555	23.246	5.028E-10	1.057388	0.48140	23.307	42.615

k448	0.40100	41.936	22.627	8.892E-10	1.057388	0.48140	22.688	41.996
2005iy	0.40246	41.911	22.603	9.095E-10	1.057746	0.48345	22.664	41.972
2005hw	0.40832	41.840	22.532	9.708E-10	1.059193	0.49168	22.595	41.903
f076	0.41000	41.347	22.039	1.528E-09	1.059610	0.49405	22.102	41.410
f096	0.41200	41.424	22.116	1.425E-09	1.060108	0.49687	22.179	41.487
04D2fp	0.41500	41.877	22.569	9.388E-10	1.060857	0.50111	22.633	41.941
1997am	0.41600	42.424	23.115	5.673E-10	1.061107	0.50253	23.180	42.488
k485	0.41600	41.558	22.249	1.260E-09	1.061107	0.50253	22.314	41.622
2005jv	0.42093	42.134	22.825	7.410E-10	1.062346	0.50952	22.891	42.199
g133	0.42100	42.136	22.828	7.393E-10	1.062365	0.50962	22.894	42.202
h342	0.42100	42.180	22.871	7.102E-10	1.062365	0.50962	22.937	42.245
f235	0.42200	41.728	22.420	1.076E-09	1.062617	0.51104	22.486	41.794
2002ab	0.42300	41.566	22.257	1.250E-09	1.062870	0.51246	22.324	41.632
1994g	0.42500	41.202	21.894	1.747E-09	1.063377	0.51531	21.961	41.269
b013	0.42600	41.762	22.453	1.044E-09	1.063631	0.51674	22.520	41.829
e148	0.42900	41.890	22.582	9.274E-10	1.064395	0.52102	22.650	41.958
04D2gb	0.43000	41.811	22.503	9.975E-10	1.064650	0.52245	22.571	41.879
1996cn	0.43000	41.802	22.494	1.006E-09	1.064650	0.52245	22.562	41.870
1996e	0.43000	41.319	22.011	1.569E-09	1.064650	0.52245	22.079	41.387
1996u	0.43000	42.399	23.090	5.806E-10	1.064650	0.52245	23.158	42.467
1997q	0.43000	41.766	22.458	1.039E-09	1.064650	0.52245	22.526	41.834
1998ba	0.43000	42.184	22.876	7.073E-10	1.064650	0.52245	22.944	42.252
d089	0.43600	41.880	22.572	9.360E-10	1.066189	0.53105	22.641	41.950
d097	0.43600	41.926	22.618	8.973E-10	1.066189	0.53105	22.687	41.995

1997ce	0.44000	42.050	22.741	8.007E-10	1.067222	0.53680	22.812	42.120
1998aw	0.44000	42.011	22.702	8.299E-10	1.067222	0.53680	22.773	42.081
03D3aw	0.44900	42.023	22.715	8.204E-10	1.069566	0.54980	22.788	42.096
1995az	0.45000	42.402	23.094	5.785E-10	1.069828	0.55125	23.168	42.476
1996cm	0.45000	42.271	22.963	6.529E-10	1.069828	0.55125	23.036	42.344
1997ai	0.45000	41.832	22.524	9.783E-10	1.069828	0.55125	22.597	41.905
04D3gt	0.45100	41.797	22.489	1.011E-09	1.070090	0.55270	22.562	41.870
1995aq	0.45300	42.821	23.513	3.933E-10	1.070616	0.55560	23.587	42.895
1999ff	0.45500	42.324	23.016	6.219E-10	1.071143	0.55851	23.090	42.399
04Yow	0.46000	42.148	22.840	7.309E-10	1.072466	0.56580	22.916	42.224
03D3cd	0.46070	42.072	22.764	7.845E-10	1.072652	0.56682	22.840	42.148
03D3cc	0.46270	42.042	22.734	8.060E-10	1.073184	0.56975	22.811	42.119
m158	0.46300	41.948	22.640	8.793E-10	1.073263	0.57018	22.716	42.025
1995ar	0.46500	41.825	22.517	9.845E-10	1.073797	0.57311	22.594	41.902
03D4au	0.46800	42.549	23.241	5.055E-10	1.074599	0.57751	23.319	42.627
e108	0.46900	42.354	23.046	6.046E-10	1.074868	0.57898	23.125	42.433
04D3df	0.47000	42.135	22.826	7.404E-10	1.075136	0.58045	22.905	42.213
1997p	0.47200	41.969	22.661	8.622E-10	1.075674	0.58339	22.740	42.048
2002dc	0.47500	42.105	22.797	7.609E-10	1.076483	0.58781	22.877	42.185
1999fn	0.47700	42.054	22.746	7.971E-10	1.077024	0.59076	22.827	42.135
1995k	0.47900	42.352	23.044	6.061E-10	1.077566	0.59372	23.125	42.433
1995ay	0.48000	42.160	22.852	7.232E-10	1.077838	0.59520	22.933	42.241
1996cg	0.49000	41.787	22.479	1.019E-09	1.080570	0.61005	22.563	41.872
g160	0.49300	42.147	22.839	7.320E-10	1.081396	0.61452	22.924	42.232

1996ci	0.49500	42.119	22.811	7.511E-10	1.081948	0.61751	22.896	42.204
h319	0.49500	42.249	22.941	6.665E-10	1.081948	0.61751	23.026	42.334
03D1ax	0.49600	42.215	22.907	6.877E-10	1.082225	0.61901	22.992	42.300
1998ax	0.49700	42.325	23.016	6.215E-10	1.082501	0.62050	23.102	42.411
e149	0.49700	42.076	22.768	7.815E-10	1.082501	0.62050	22.854	42.162
1995as	0.49800	42.984	23.676	3.387E-10	1.082778	0.62200	23.762	43.070
1997cj	0.50000	42.363	23.055	5.999E-10	1.083333	0.62500	23.142	42.450
03D1au	0.50430	42.338	23.030	6.137E-10	1.084531	0.63146	23.118	42.426
p524	0.50800	42.194	22.886	7.007E-10	1.085565	0.63703	22.975	42.283
g120	0.51000	41.881	22.573	9.354E-10	1.086126	0.64005	22.662	41.970
2001gy	0.51100	42.374	23.066	5.940E-10	1.086407	0.64156	23.155	42.464
2002ad	0.51400	42.786	23.478	4.062E-10	1.087251	0.64610	23.569	42.877
d084	0.51900	43.072	23.763	3.124E-10	1.088664	0.65368	23.856	43.164
04D2gc	0.52100	42.180	22.872	7.099E-10	1.089231	0.65672	22.965	42.273
05Zwi	0.52100	42.378	23.070	5.915E-10	1.089231	0.65672	23.163	42.471
n258	0.52200	42.677	23.369	4.491E-10	1.089515	0.65824	23.462	42.770
04D1ak	0.52600	42.400	23.092	5.797E-10	1.090654	0.66434	23.186	42.494
1997h	0.52600	41.956	22.648	8.726E-10	1.090654	0.66434	22.742	42.050
2001jp	0.52800	42.454	23.146	5.515E-10	1.091225	0.66739	23.241	42.549
n285	0.52800	42.370	23.062	5.962E-10	1.091225	0.66739	23.156	42.464
03D3af	0.53200	42.566	23.258	4.977E-10	1.092371	0.67351	23.354	42.662
f011	0.53900	42.227	22.919	6.801E-10	1.094386	0.68426	23.016	42.325
1997eq	0.54000	42.425	23.116	5.668E-10	1.094675	0.68580	23.215	42.523
f244	0.54000	42.511	23.203	5.234E-10	1.094675	0.68580	23.301	42.609

2000fr	0.54300	42.479	23.171	5.392E-10	1.095544	0.69042	23.270	42.578
03D1gt	0.54800	42.296	22.988	6.379E-10	1.096997	0.69815	23.089	42.397
04D4bq	0.55000	42.276	22.967	6.501E-10	1.097581	0.70125	23.069	42.377
1997l	0.55000	44.343	25.035	9.680E-11	1.097581	0.70125	25.136	44.445
04D3hn	0.55160	42.303	22.995	6.339E-10	1.098048	0.70373	23.097	42.405
2001go	0.55200	42.511	23.203	5.235E-10	1.098165	0.70435	23.304	42.613
04D1ag	0.55700	42.557	23.249	5.017E-10	1.099630	0.71212	23.352	42.660
f041	0.56100	42.872	23.564	3.754E-10	1.100807	0.71836	23.668	42.976
m034	0.56200	43.051	23.742	3.185E-10	1.101102	0.71992	23.847	43.155
k411	0.56400	42.373	23.065	5.944E-10	1.101693	0.72305	23.170	42.478
2001iy	0.56800	42.709	23.401	4.361E-10	1.102878	0.72931	23.507	42.816
1996cf	0.57000	42.671	23.363	4.515E-10	1.103471	0.73245	23.470	42.778
1996i	0.57000	42.489	23.181	5.341E-10	1.103471	0.73245	23.288	42.596
03D4gl	0.57100	42.399	23.091	5.802E-10	1.103769	0.73402	23.198	42.506
1997af	0.57900	43.186	23.878	2.810E-10	1.106156	0.74662	23.988	43.296
1997f	0.58000	43.305	23.997	2.519E-10	1.106456	0.74820	24.107	43.415
03D4gf	0.58100	42.742	23.434	4.230E-10	1.106756	0.74978	23.544	42.852
1997aj	0.58100	42.063	22.755	7.905E-10	1.106756	0.74978	22.865	42.174
m138	0.58100	43.669	24.361	1.802E-10	1.106756	0.74978	24.471	43.779
03D1aw	0.58170	42.672	23.364	4.513E-10	1.106966	0.75089	23.474	42.782
k430	0.58200	43.164	23.856	2.869E-10	1.107056	0.75136	23.966	43.274
d058	0.58300	42.401	23.093	5.792E-10	1.107356	0.75294	23.204	42.512
b010	0.59100	43.209	23.901	2.752E-10	1.109768	0.76564	24.014	43.322
03D4gg	0.59200	42.577	23.269	4.924E-10	1.110070	0.76723	23.383	42.691

1997k	0.59200	44.152	24.844	1.154E-10	1.110070	0.76723	24.958	44.266
f216	0.59900	42.743	23.435	4.225E-10	1.112195	0.77840	23.551	42.859
h323	0.60300	42.646	23.338	4.621E-10	1.113415	0.78480	23.455	42.763
03D4dy	0.60400	42.527	23.219	5.158E-10	1.113721	0.78641	23.336	42.644
04D3do	0.61000	42.905	23.597	3.641E-10	1.115559	0.79605	23.716	43.024
e138	0.61200	42.814	23.506	3.960E-10	1.116174	0.79927	23.625	42.933
04D4an	0.61300	42.994	23.686	3.356E-10	1.116481	0.80088	23.805	43.113
p534	0.61300	42.617	23.309	4.749E-10	1.116481	0.80088	23.428	42.736
1995ax	0.61500	42.543	23.234	5.084E-10	1.117098	0.80411	23.355	42.663
f231	0.61900	43.056	23.748	3.169E-10	1.118333	0.81058	23.869	43.177
04D3co	0.62000	43.009	23.701	3.308E-10	1.118642	0.81220	23.823	43.131
1996h	0.62000	43.228	23.920	2.704E-10	1.118642	0.81220	24.042	43.350
Z-005	0.62300	42.515	23.206	5.217E-10	1.119571	0.81706	23.329	42.637
03D4dh	0.62680	42.759	23.450	4.167E-10	1.120752	0.82324	23.574	42.882
e140	0.63100	42.377	23.069	5.920E-10	1.122060	0.83008	23.194	42.502
n256	0.63100	42.882	23.574	3.720E-10	1.122060	0.83008	23.699	43.007
03D4at	0.63300	43.050	23.742	3.187E-10	1.122685	0.83334	23.867	43.176
g050	0.63300	42.202	22.894	6.960E-10	1.122685	0.83334	23.019	42.327
1998ay	0.64000	43.164	23.856	2.868E-10	1.124878	0.84480	23.984	43.292
1998be	0.64000	42.764	23.456	4.145E-10	1.124878	0.84480	23.584	42.892
2003be	0.64000	42.924	23.616	3.577E-10	1.124878	0.84480	23.744	43.052
04D3cy	0.64300	43.011	23.703	3.302E-10	1.125821	0.84972	23.832	43.140
e147	0.64500	42.817	23.509	3.949E-10	1.126451	0.85301	23.638	42.946
1995at	0.65500	42.315	23.007	6.269E-10	1.129615	0.86951	23.139	42.447

1996ck	0.65600	43.145	23.837	2.920E-10	1.129932	0.87117	23.969	43.277
1997r	0.65700	42.975	23.667	3.413E-10	1.130250	0.87282	23.800	43.108
2003bd	0.67000	43.143	23.835	2.924E-10	1.134401	0.89445	23.972	43.280
2001gq	0.67100	42.982	23.674	3.392E-10	1.134722	0.89612	23.811	43.119
03D1co	0.67900	43.457	24.149	2.189E-10	1.137296	0.90952	24.289	43.597
k441	0.68000	42.904	23.596	3.645E-10	1.137619	0.91120	23.736	43.044
g240	0.68700	42.996	23.688	3.347E-10	1.139884	0.92298	23.830	43.139
h300	0.68700	42.835	23.527	3.884E-10	1.139884	0.92298	23.669	42.977
03D1fl	0.68800	43.054	23.746	3.174E-10	1.140209	0.92467	23.888	43.196
04D2iu	0.69100	43.089	23.781	3.074E-10	1.141183	0.92974	23.924	43.232
03D4cz	0.69500	43.212	23.904	2.745E-10	1.142485	0.93651	24.048	43.356
2001jb	0.69800	43.769	24.461	1.644E-10	1.143464	0.94160	24.606	43.914
04D2gp	0.70700	43.282	23.974	2.572E-10	1.146412	0.95692	24.123	43.431
04D3is	0.71000	43.022	23.714	3.269E-10	1.147398	0.96205	23.863	43.171
2001ix	0.71100	43.567	24.258	1.980E-10	1.147727	0.96376	24.408	43.716
04D1aj	0.72100	43.176	23.868	2.838E-10	1.151029	0.98092	24.020	43.328
04D3fq	0.73000	43.277	23.969	2.585E-10	1.154017	0.99645	24.124	43.433
2002kd	0.73500	43.092	23.784	3.066E-10	1.155684	1.00511	23.941	43.249
04Rak	0.74000	43.288	23.979	2.560E-10	1.157356	1.01380	24.138	43.446
04D2ja	0.74100	43.717	24.409	1.723E-10	1.157691	1.01554	24.568	43.876
1998bi	0.75000	43.243	23.935	2.667E-10	1.160714	1.03125	24.097	43.405
04D3ks	0.75200	43.302	23.994	2.527E-10	1.161388	1.03475	24.156	43.464
04D3oe	0.75600	43.814	24.506	1.576E-10	1.162738	1.04177	24.670	43.978
1997g	0.76300	44.472	25.164	8.596E-11	1.165107	1.05408	25.330	44.638

2001fo	0.77200	43.510	24.202	2.086E-10	1.168167	1.06999	24.371	43.679
1997ez	0.78000	43.601	24.293	1.918E-10	1.170899	1.08420	24.464	43.772
p528	0.78100	43.437	24.129	2.230E-10	1.171241	1.08598	24.301	43.609
03D4fd	0.79100	43.577	24.269	1.961E-10	1.174674	1.10384	24.444	43.752
2001hx	0.79900	43.379	24.071	2.354E-10	1.177432	1.11820	24.248	43.556
03D1fq	0.80000	43.714	24.406	1.728E-10	1.177778	1.12000	24.584	43.892
04D3ny	0.81000	43.370	24.062	2.373E-10	1.181243	1.13805	24.243	43.551
04D4dm	0.81100	43.405	24.096	2.299E-10	1.181591	1.13986	24.278	43.586
2001hy	0.81200	43.654	24.345	1.827E-10	1.181938	1.14167	24.527	43.835
2001jf	0.81500	44.074	24.766	1.240E-10	1.182982	1.14711	24.949	44.257
1999fj	0.81600	43.691	24.383	1.765E-10	1.183330	1.14893	24.566	43.874
04D3nc	0.81700	43.652	24.343	1.831E-10	1.183679	1.15074	24.526	43.835
03D4cn	0.81800	43.393	24.085	2.323E-10	1.184028	1.15256	24.268	43.576
P-009	0.82100	43.641	24.333	1.849E-10	1.185074	1.15802	24.517	43.825
04D3lu	0.82180	43.815	24.507	1.574E-10	1.185354	1.15948	24.692	44.000
04D3cp	0.83000	44.051	24.743	1.268E-10	1.188224	1.17445	24.930	44.238
1997ap	0.83000	43.542	24.234	2.024E-10	1.188224	1.17445	24.421	43.730
2001hs	0.83300	43.688	24.380	1.770E-10	1.189277	1.17994	24.568	43.876
05Spo	0.83900	43.398	24.090	2.312E-10	1.191387	1.19096	24.280	43.588
04D4bk	0.84000	43.873	24.565	1.493E-10	1.191739	1.19280	24.755	44.064
2003eq	0.84000	43.514	24.206	2.077E-10	1.191739	1.19280	24.397	43.705
H-003	0.85000	43.494	24.186	2.116E-10	1.195270	1.21125	24.380	43.688
04Man	0.85400	43.608	24.300	1.905E-10	1.196687	1.21866	24.495	43.803
2002x	0.85900	44.093	24.785	1.219E-10	1.198462	1.22794	24.981	44.289

1997ek	0.86000	43.922	24.614	1.428E-10	1.198817	1.22980	24.810	44.119
03D1ew	0.86800	43.493	24.185	2.118E-10	1.201666	1.24471	24.385	43.693
03D1cm	0.87000	44.231	24.923	1.073E-10	1.202380	1.24845	25.123	44.431
2001fs	0.87400	43.295	23.987	2.542E-10	1.203809	1.25594	24.189	43.497
2001hu	0.88200	43.375	24.067	2.362E-10	1.206675	1.27096	24.271	43.579
2001jh	0.88500	44.184	24.876	1.121E-10	1.207752	1.27661	25.081	44.389
03D4di	0.90500	43.639	24.331	1.853E-10	1.214967	1.31451	24.542	43.850
04D3gx	0.91000	44.306	24.997	1.002E-10	1.216780	1.32405	25.210	44.519
03D4cy	0.92710	43.940	24.632	1.403E-10	1.223007	1.35686	24.851	44.159
04D3ki	0.93000	43.552	24.244	2.006E-10	1.224067	1.36245	24.464	43.772
2003XX	0.93500	43.540	24.232	2.029E-10	1.225898	1.37211	24.453	43.761
2001kd	0.93600	43.307	23.999	2.514E-10	1.226264	1.37405	24.220	43.529
03D4cx	0.94900	43.445	24.137	2.215E-10	1.231042	1.39930	24.362	43.671
04D3ml	0.95000	43.977	24.668	1.357E-10	1.231410	1.40125	24.894	44.203
1999fm	0.95000	43.638	24.330	1.853E-10	1.231410	1.40125	24.556	43.864
2002dd	0.95000	43.884	24.576	1.478E-10	1.231410	1.40125	24.802	44.110
2001cw	0.95300	44.274	24.965	1.032E-10	1.232516	1.40710	25.192	44.501
04D3nr	0.96000	43.613	24.305	1.897E-10	1.235102	1.42080	24.534	43.842
04D4dw	0.96100	44.264	24.956	1.041E-10	1.235472	1.42276	25.186	44.494
04Pat	0.97000	44.463	25.154	8.674E-11	1.238807	1.44045	25.387	44.695
1997ck	0.97000	42.821	23.513	3.934E-10	1.238807	1.44045	23.746	43.054
C-001	0.97400	43.834	24.526	1.547E-10	1.240293	1.44834	24.760	44.068
04Omb	0.97500	44.334	25.026	9.767E-11	1.240665	1.45031	25.260	44.568
2001jm	0.97800	43.500	24.192	2.105E-10	1.241781	1.45624	24.427	43.735

04D3lp	0.98300	44.157	24.849	1.149E-10	1.243643	1.46614	25.086	44.394
04D3dd	1.01000	44.012	24.704	1.313E-10	1.253756	1.52005	24.950	44.258
05Str	1.01000	44.912	25.604	5.733E-11	1.253756	1.52005	25.850	45.158
D-000	1.01700	44.294	24.986	1.013E-10	1.256393	1.53414	25.234	44.542
04Eag	1.02000	44.363	25.055	9.508E-11	1.257525	1.54020	25.304	44.612
05Fer	1.02000	44.164	24.856	1.142E-10	1.257525	1.54020	25.105	44.413
2001hb	1.03000	44.240	24.932	1.065E-10	1.261305	1.56045	25.184	44.492
1999fk	1.05700	44.151	24.842	1.156E-10	1.271572	1.61562	25.103	44.411
C-000	1.09200	44.008	24.700	1.319E-10	1.285006	1.68823	24.972	44.280
F-012	1.11000	44.625	25.317	7.467E-11	1.291967	1.72605	25.595	44.903
05Gab	1.12000	44.514	25.206	8.270E-11	1.295849	1.74720	25.488	44.796
2001gn	1.12400	44.568	25.259	7.875E-11	1.297405	1.75569	25.542	44.850
04Gre	1.14000	44.323	25.015	9.865E-11	1.303645	1.78980	25.303	44.611
2002ki	1.14000	44.197	24.889	1.108E-10	1.303645	1.78980	25.177	44.485
N-033	1.18800	44.608	25.299	7.589E-11	1.322519	1.89367	25.603	44.911
05Red	1.19000	44.363	25.055	9.509E-11	1.323311	1.89805	25.359	44.667
A-004	1.19200	44.459	25.151	8.705E-11	1.324102	1.90243	25.455	44.764
R-012	1.21500	45.247	25.938	4.214E-11	1.333234	1.95311	26.251	45.559
05Lan	1.23000	45.021	25.713	5.188E-11	1.339215	1.98645	26.030	45.338
H-005	1.24100	44.582	25.274	7.773E-11	1.343615	2.01104	25.594	44.902
2003az	1.26500	44.944	25.636	5.567E-11	1.353251	2.06511	25.964	45.273
2002fw	1.30000	45.016	25.708	5.209E-11	1.367391	2.14500	26.048	45.356
2002hp	1.30500	44.740	25.432	6.717E-11	1.369420	2.15651	25.773	45.082
2003aj	1.30700	45.411	26.103	3.622E-11	1.370232	2.16112	26.445	45.753

D-006	1.31500	44.971	25.663	5.429E-11	1.373483	2.17961	26.008	45.316
2003dy	1.34000	45.068	25.759	4.969E-11	1.383675	2.23780	26.112	45.420
G-004	1.35000	44.827	25.519	6.201E-11	1.387766	2.26125	25.875	45.183
04Mcg	1.37000	45.050	25.742	5.051E-11	1.395970	2.30845	26.104	45.412
04Sas	1.39000	44.876	25.568	5.926E-11	1.404205	2.35605	25.937	45.245
K-000	1.41400	44.804	25.496	6.335E-11	1.414125	2.41370	25.872	45.180

Table S1 legend

Data in Supplemental Table S1 are presented with a set number of significant digits, however all information was carried forward through the analyses described below.

SN: The names of SNe, provided in the SCP Union 2.1 compilation [53,54].

***z*:** The observed redshift of SNe, provided in the SCP Union 2.1 compilation.

***m-M*:** Distance modulus (*m-M*) of SNe, provided in the SCP Union 2.1 compilation.

***m*:** The apparent magnitude (*m*) was calculated by adding the absolute magnitude *M* to the distance modulus (*m-M*):

$$m = m\text{-}M + M \qquad \text{(S1)}$$

The value of *M* (with systematics) is from the SCP Union 2.1 compilation: -19.3081547178.

flux: The observed flux of SNe (f_x/f_{x0}) was calculated from the apparent magnitude. The formula for apparent magnitude

$$m = -2.5\log_{10}\left(\frac{f_x}{f_{x0}}\right) \qquad (12)$$

was restated to solve for f_x/f_{x0}:

$$\frac{f_x}{f_{xO}} = \frac{1}{10^{(2m/5)}} \qquad \text{(S2)}$$

***TC*:** The time contraction ratio (*TC*) for SNe Ia relative to present time was calculated using equation (6), the derivation of which is described in the text.

$$TC = \frac{1}{\sqrt{1 - \left(\frac{(1+z)^2 - 1}{(1+z)^2 + 1} \right)^2}} \tag{6}$$

z_{TC}: The time-contracted redshift for SNe was calculated using equation (11), the derivation of which is described in the text.

$$z_{TC} = TC(1+z) - 1 \tag{11}$$

m_{TC}: The apparent magnitude compensated for time contraction effects was calculated using equation (13), the derivation of which is described in the text.

$$m_{TC} = -2.5 \log_{10} \left(\frac{1}{TC} \cdot \frac{f_x}{f_{x0}} \right) \tag{13}$$

$m\text{-}M_{TC}$: Distance modulus (*m-M*) adjusted to compensate for time-contraction, was calculated by subtracting absolute magnitude (*M*) from the apparent magnitude adjusted to compensate for time contraction effects (m_{TC}):

$$m\text{-}M_{TC} = m_{TC} - M \tag{S3}$$
